# *Salmonella* surveillance in raw pet food and dogs in Great Britain, 2013–2022

**DOI:** 10.3389/fvets.2026.1750907

**Published:** 2026-02-25

**Authors:** Lucy C. Snow, Andrew D. Wales, Susan M. Withenshaw, Joanna R. Lawes, Adrienne Mackintosh, Francesca Martelli

**Affiliations:** 1 Department of Epidemiological Sciences, Animal and Plant Health Agency, Addlestone, United Kingdom; 2 Department of Comparative Biomedical Sciences, Faculty of Health and Medical Sciences, School of Veterinary Medicine, University of Surrey, Guilford, United Kingdom; 3 Department of Bacteriology, Animal and Plant Health Agency, Addlestone, United Kingdom

**Keywords:** antimicrobial resistance, dogs, foodborne pathogen, raw meat pet food, *Salmonella*, zoonoses

## Abstract

**Background:**

Raw meat pet food is increasingly popular in Great Britain prompting concerns about its potential to transmit zoonotic pathogens, particularly *Salmonella*.

**Aim:**

To investigate correlations between *Salmonella* isolated from dog food (both raw and heat-treated) and from dogs, using historical passive surveillance data from manufacturing plants and clinical samples.

**Methods:**

Data collected by the Animal and Plant Health Agency from 2013 to 2022 (phenotypically-determined *Salmonella* serovars plus their phage types and disc-diffusion antimicrobial resistance profiles) were compared between dog food and canine clinical sources.

**Results:**

Over time both the number and serovar diversity of *Salmonella* isolations from raw meat pet food increased, from 4 isolates of 4 serovars in 2013 to 606 isolates of 39 serovars in 2022, in parallel with a five-fold increase in the number of raw meat pet food plants operating in Great Britain. Between 2021 and 2022, following the implementation of statutory *Salmonella* reporting in dogs, considerable overlaps in serovar distributions were observed between raw meat pet food and dog samples, with serovars of significant public health importance such as *S.* Typhimurium, monophasic *S.* Typhimurium and *S.* Infantis among the top 10 most frequently isolated from both sources. Some serovars, like *S.* Indiana and subspecies *diarizonae*, were more frequently isolated from raw meat pet food while others like *S.* Typhimurium and *S.* Dublin, were over twice as frequent among dog isolates. Antimicrobial resistance patterns revealed some correlations between sources for certain serovars, such as *S.* Typhimurium, while for others (including *S*. Dublin) resistance patterns were unique to the dog isolates. Resistances to cefotaxime, ceftazidime or ciprofloxacin were rare, although exceptionally 9.9% of *S.* Infantis isolates from raw meat pet food showed ciprofloxacin resistance. *S*. Kentucky resistant to cefotaxime, ceftazidime and ciprofloxacin was isolated from a dog sample.

**Conclusion:**

Despite limitations for establishing direct transmission pathways, the findings highlight raw meat pet food as a potential vector for *Salmonella* transmission, emphasizing the risks to both animal and public health and underlining the need for vigilant monitoring and hygiene practices. For antimicrobial resistance risk, generally high susceptibility to the extended spectrum cephalosporin and fluoroquinolone classes is reassuring, although the detection of multi-drug-resistant strains highlights ongoing concerns.

## 1 Introduction

There were over 8,100 reported cases of human salmonellosis in England in 2022, with an estimated 4.7 unreported community infections for each documented case (1, 2). Human salmonellosis is the second most frequently reported zoonosis in the United Kingdom (UK), and in Europe more widely, and the condition is associated with a notably high proportion of hospitalized cases in comparison with other major zoonotic diseases (3).

*Salmonella* spp. can be carried and shed in feces by dogs (*Canis lupus familiaris*) following its ingestion in food, often without accompanying clinical signs (4–7). Epidemiological links have also been demonstrated between human cases of salmonellosis and *Salmonella* contamination of pet food or pet treats (4, 8–11). Contamination of pet food by *Salmonella* is therefore a concern for public and veterinary health, as well as for biosecurity when *Salmonella*-shedding dogs have access to land used by livestock (12, 13). *Salmonella* incidents can occur with conventional heat-treated compounded pet food when, for example, a handling facility downstream of the thermal microbiological kill stage is contaminated (8). However, there may be increased risk for raw food (12) and dried treats (14) because this material is never subject to a heat critical control step to reduce bacterial load.

Raw feeding of pet dogs and cats has become increasingly popular in Great Britain and elsewhere in recent years, with strong growth in the commercial preparation and sale of raw pet food being evident in market analysis (15) and in registrations of raw food manufacturing premises (16). Marketing of such products leads many consumers to perceive raw feeding as healthier and more “natural” for pets than using heat-processed foods. However, evidence is largely anecdotal, or of limited scientific quality (17) or uncertain significance (18, 19). There are no prospective studies which evaluate raw feeding claims while adjusting for bias in data gathering and measures of outcome.

In Great Britain, animal by-products (ABP) used in all pet food manufacture are governed by regulations that constrain sources to animal tissues deemed fit for human consumption, with some closely defined exceptions (20). Imports of such material for the same use, or of prepared raw food for direct sale, are restricted to products coming from specified countries or sub-regions, and from establishments approved by Great Britain that use equivalent classifications of ABP (21, 22). ABP regulations require random testing of products for *Salmonella* at all premises in Great Britain approved for manufacturing non-canned pet food (20). Sampling regimes are developed with, and overseen by, the Animal and Plant Health Agency (APHA) in Great Britain and vary according to throughput, variety of products, batch size and type of animal-derived products used. Typically, pools of ten 30 g sub-samples are taken at random from each product line at a frequency (for example, weekly or monthly) reflecting production volume. All samples testing positive for *Salmonella* are required under the UK Zoonoses Order 1989 to be submitted to APHA for confirmation.

In 2021 the UK Zoonoses Order 1989 was amended to mandate the reporting of *Salmonella* isolations from dogs, thus making surveillance statutory when it had previously been voluntary. This came into force in February in England and in April in Scotland and Wales and has led to increased numbers of *Salmonella* isolates from clinical dog submissions being available for microbiological and statistical analysis (23). Thus, more meaningful comparisons are now possible between *Salmonella* strains in animal feed and in clinical (usually fecal) samples from dogs, albeit from passive surveillance that would not include most sub-clinically affected animals.

Here we summarize APHA surveillance data of clinical *Salmonella* isolations from dogs between 2013 and 2022, with an emphasis on the period of statutory reporting from February 2021 onwards. We also describe trends in *Salmonella* isolated from samples of raw meat pet food (RMPF) as a result of statutory ABP testing between 2013 and 2022, in an attempt to understand whether associations may exist in the occurrence of *Salmonella* in RMPF and in dogs.

## 2 Materials and methods

### 2.1 Raw pet food manufacturing plants

Data on the number of manufacturing plants that were approved and registered by APHA to produce raw pet food using animal by products were obtained from the System for Recording Animal-By-Product Information (SRABPI) run by APHA for the years 2013–2022 (24). Only plants where the final product was listed as raw pet food were included. A plant was defined as a unique combination of postcode plus owner.

### 2.2 Pet food

Data were obtained from the APHA *Salmonella* database (*Salmonella* serovar, phage type, antimicrobial resistance profiles and associated epidemiological details) on all feed submissions to APHA collected between 2013 and 2022. Feed submissions to APHA come primarily from private laboratories rather than direct from producers and are accompanied by a standard submission form (25) designed to collect statutory information including the nature of the product sampled and species for which the feed is intended. There is no dedicated “Raw pet food” category on the APHA *Salmonella* submission form, making consistent identification of these samples challenging due to variation in how raw pet food is recorded by submitters. As a result, it can be difficult to distinguish between finished raw pet food products and samples taken from pet food ingredients that may have been diverted for heat treatment or discarded, following a positive *Salmonella* result. It is also not possible to determine what proportion of *Salmonella* positive raw pet food ingredients included in this study ultimately entered finished products.

#### 2.2.1 Definitions and classification of pet food samples

For the purposes of the current analysis, the two mutually exclusive categories of “RMPF” and “compound feed” were defined as follows:

Raw meat pet food (RMPF): samples initially submitted as “Raw material” or “Other” were classified as raw pet food where additional information provided by submitters supported this, including cases where samples originated from specialist raw pet food producers. Samples for which submission details indicated other feed types were reviewed and excluded if necessary. Submissions containing insect or fish-based ingredients, or food intended for cats were also removed. The included samples therefore predominantly represent RMPF intended for dogs.

Compound dog food: samples submitted initially as “Compound Feed,” where producer and additional information supported this, i.e., it did not indicate raw pet food, and species for which the feed was intended was “Dog.” Even though thermal treatment is not a declared category in the database, heat processing is characteristic of production methods for the majority of compound dog food in the UK.

### 2.3 Dogs

Data on clinical *Salmonella* isolates from dogs were obtained from the APHA *Salmonella* database for the years 2013–2022. Isolates from dogs listed as imported were removed from the dataset.

### 2.4 Strain characterization

Between 2013 and 2022, prior to the adoption of Whole Genome Sequencing as the primary method of *Salmonella* serotyping, all *Salmonella* isolates from animals and feed at APHA were phenotypically serotyped by micro-, tube and/or slide agglutination tests, and serovars were derived by reference to the White-Kauffmann-Le Minor Scheme (26). Serotyping for all years was carried out at the APHA national reference laboratory for *Salmonella* which is UKAS-accredited to BS EN ISO 17025 for an extensive range of tests, supported by proficiency testing accredited to BS ISO 17043. Isolates of *Salmonella enterica* subsp. *enterica* serovars Typhimurium and Enteritidis were phage-typed according to the UKHSA, Colindale and Ward schemes (27, 28). These are the only serovars routinely phage typed by APHA, due in part to their public health importance and to align with methodology employed by UK public health partners. All *Salmonella* isolates were also tested by disc diffusion for their sensitivity *in vitro* to 16 antimicrobials (29). The choice of antimicrobials, which is reviewed periodically, is designed to provide a core set of those used in veterinary and human medicine (30). There were no major changes to strain characterization methods between 2013 and 2022, which might otherwise have affected interpretation of the data.

### 2.5 Data processing

Data were cleaned and manipulated using the statistical software STATA 15 (StataCorp LLC, Texas, United States) and Excel 365 (Microsoft Corporation, Washington State, United States).

## 3 Results

### 3.1 Raw pet food manufacturing plants

**Figure 1:**
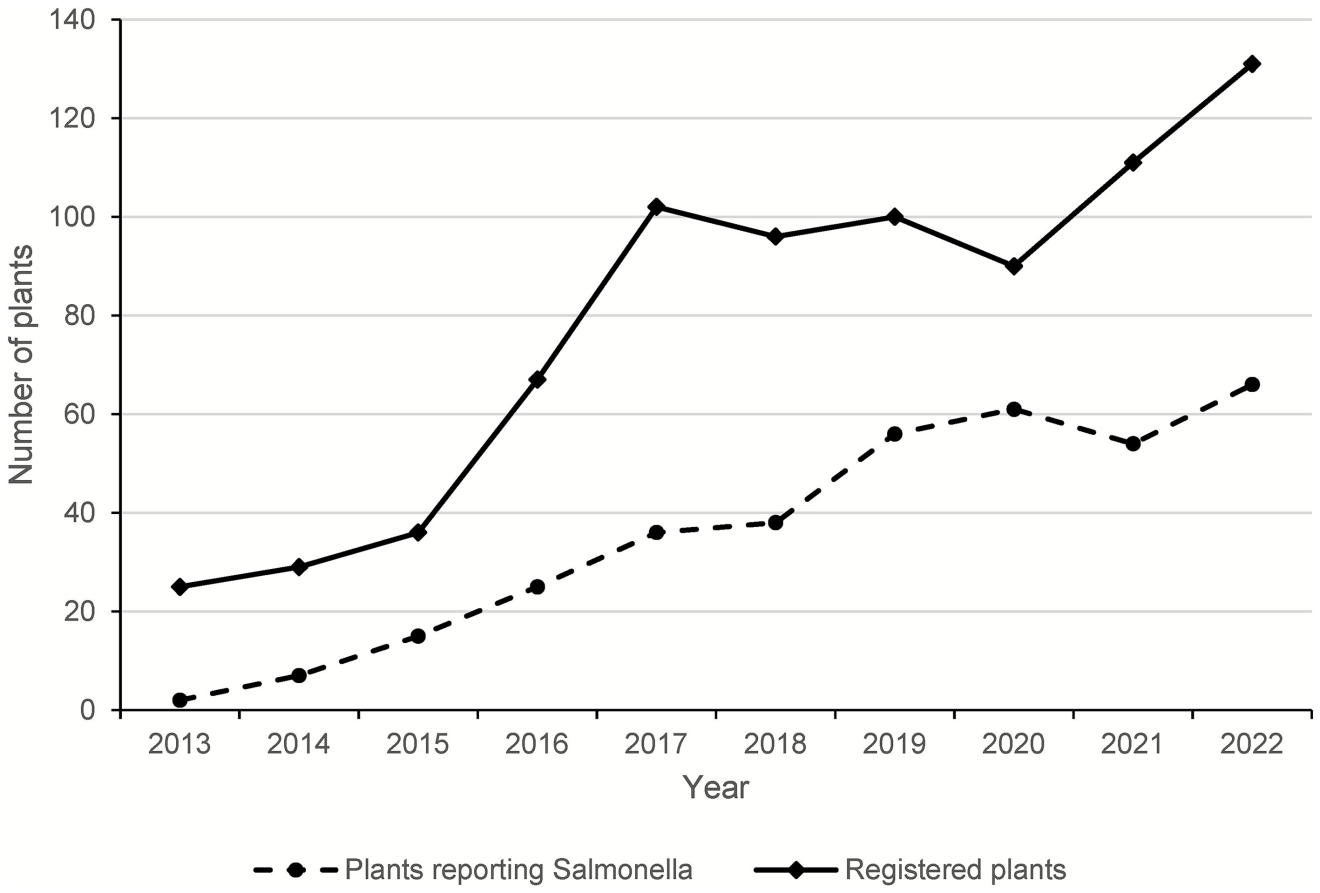
The number of plants approved to produce raw pet food and the number isolating *Salmonella*, 2013–2022.

The number of manufacturing plants registered and approved by APHA to produce raw pet food increased five-fold, from 25 in 2013 to 131 in 2022 (Figure 1). Over this period, 236 different plants were registered. The duration of operation for individual plants ranged from one to 10 years, with a median value of 3 years. The number of plants reporting *Salmonella* isolations over the same time increased from 2/25 (8%) in 2013 to 62/131 (47%) in 2022.

### 3.2 *Salmonella* in compound and raw meat pet food

**Figure 2:**
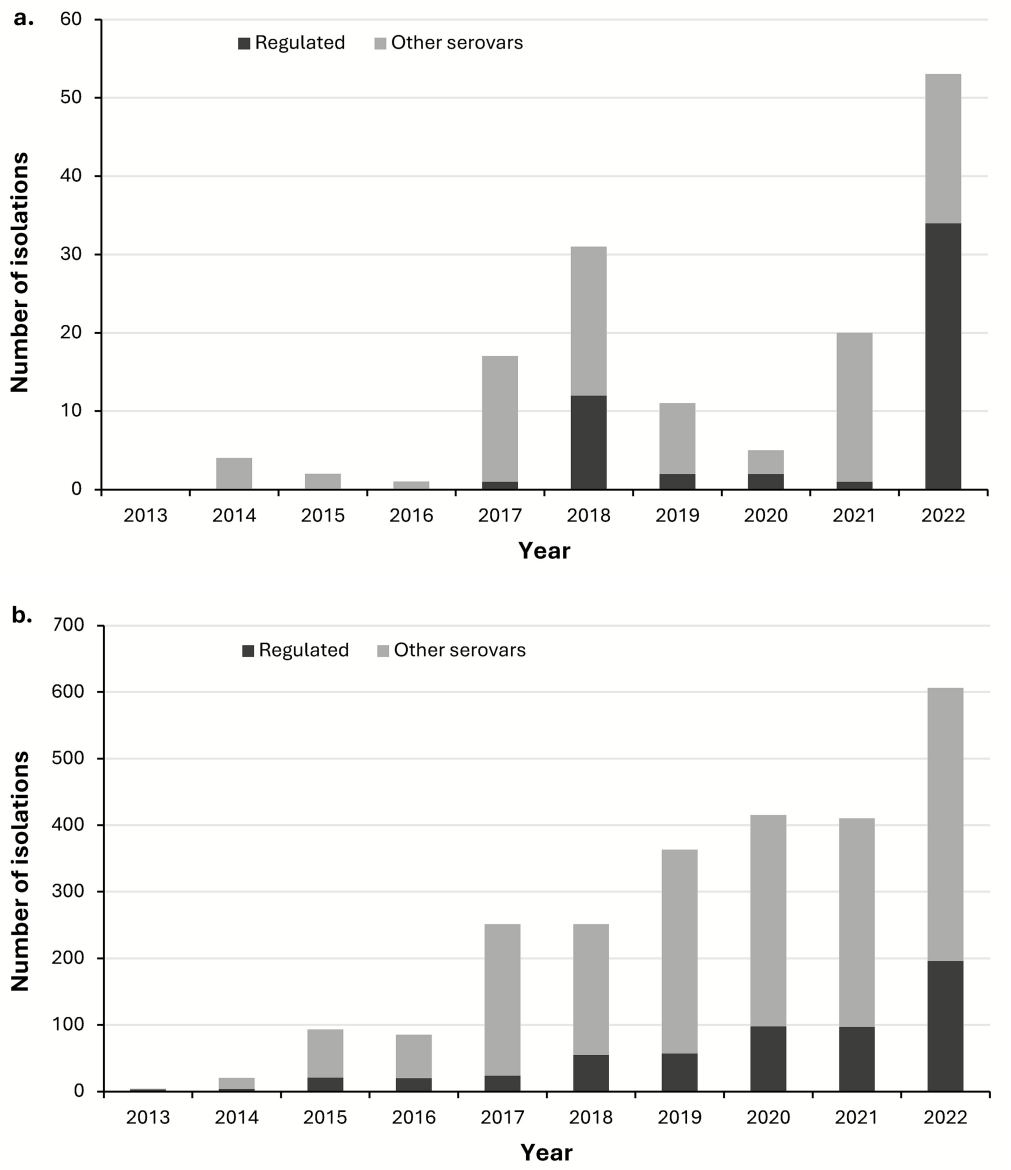
The number of *Salmonella* isolations and proportion of “regulated” and other serovars 2013–2022 in **(a)** compound dog food and **(b)** RMPF between 2013 and 2022. Regulated serovars are those regulated in the livestock sector by EU and UK law: *Salmonella enterica* subsp. *enterica* serovars: Enteritidis, Typhimurium (including monophasic variants), Infantis, Hadar, and Virchow.

There were 4,330 isolations of *Salmonella* from all animal feedstuffs and other ABP submitted to the APHA between 2013 and 2022. Of these, approximately 2,498 were from RMPF and 144 were from compound dog food. The number of *Salmonella* isolations from RMPF increased steadily during this period, whereas isolations from compound dog food were more variable and much less frequent, with a maximum of 53 in 2022 compared to 606 isolations from raw pet food in the same year (Figure 2). Certain *Salmonella* serovars are targeted for control in livestock sectors in EU law (and latterly assimilated into UK law) due to their being of significant public health importance. Currently these are: *Salmonella* Enteritidis, *S.* Typhimurium, monophasic *S.* Typhimurium variants (*S.* 4,12:i:- and *S.* 4,5,12:i:-), *S.* Infantis, *S.* Hadar and *S.* Virchow. Isolations of these “regulated” serovars varied in number from year to year but accounted for no more than one quarter of isolations in RMPF in most years, with a high of 32% observed in 2022 (Figure 2). Among isolates from compound dog food these serovars accounted for less than 6% of isolations in seven of the 10 years examined but were over 39% of isolates in 2018, 2020 and 2022.

### 3.3 Strain diversity in raw meat pet food and dogs

#### 3.3.1 Serotype and phage types

Seventy-four different serovars (not including partial structures and rough strains) were identified from RMPF during the 10 surveyed years, with the annual number ranging from four in 2013 to 39 in 2022 (Table 1). The most common serovar overall was *S.* Indiana, followed by *S.* Typhimurium, *S.* Derby and *S. enterica* subspecies *diarizonae* (Table 1). Annual variations in relative serovar frequencies typically were minor with serovars like *S.* Indiana, *S.* Typhimurium and *S.* Derby regularly featuring in the top five most common in a given year. In 2022 there were twice as many isolations of *S.* Indiana (17% of total isolations that year) as *S.* Typhimurium (8%). There was also a notably high proportion of *S.* Infantis (11%) compared to previous years when this serovar was sometimes not reported. The most frequent phage types of *S.* Typhimurium and monophasic *S.* Typhimurium were DT104 and DT193, respectively (Supplementary Table 1).

**Table 1:** *Salmonella* serovars isolated from raw meat pet food in Great Britain intended for dogs 2013 to 2022, top 20 named, listed in order of frequency.

Serovar	2013	2014	2015	2016	2017	2018	2019	2020	2021	2022	Totals
Indiana	–	1 (5%)	3 (3%)	7 (8%)	48 (19%)	25 (10%)	45 (12%)	48 (12%)	60 (15%)	101 (17%)	338
Typhimurium	1 (25%)	1 (5%)	10 (11%)	1 (1%)	4 (2%)	31 (12%)	12 (3%)	36 (9%)	35 (9%)	50 (8%)	181
Derby	–	–	5 (5%)	7 (8%)	13 (5%)	23 (9%)	19 (5%)	31 (7%)	19 (5%)	37 (6%)	154
*diarizonae* ^†^	–	–	–	1 (1%)	5 (2%)	8 (3%)	29 (8%)	56 (13%)	18 (4%)	26 (4%)	143
Mbandaka	–	–	9 (10%)	9 (11%)	9 (4%)	22 (9%)	24 (7%)	22 (5%)	23 (6%)	16 (3%)	134
4,12:i:-	–	3 (15%)	5 (5%)	10 (12%)	12 (5%)	9 (4%)	18 (5%)	17 (4%)	17 (4%)	40 (7%)	131
Kottbus	1 (25%)	1 (5%)	12 (13%)	12 (14%)	11 (4%)	14 (6%)	10 (3%)	13 (3%)	28 (7%)	22 (4%)	124
Infantis	1 (25%)	–	2 (2%)	–	8 (3%)	4 (2%)	–	16 (4%)	26 (6%)	65 (11%)	122
4,5,12:i:-	1 (25%)	–	2 (2%)	8 (9%)	–	11 (4%)	17 (5%)	10 (2%)	15 (4%)	28 (5%)	92
Newport	–	–	–	–	9 (4%)	10 (4%)	11 (3%)	11 (3%)	18 (4%)	24 (4%)	83
Montevideo	–	1 (5%)	–	8 (9%)	15 (6%)	12 (5%)	11 (3%)	6 (1%)	8 (2%)	6 (1%)	67
Give	–	1 (5%)	4 (4%)	1 (1%)	4 (2%)	7 (3%)	13 (4%)	14 (3%)	9 (2%)	11 (2%)	64
Dublin	–	–	–	2 (2%)	1 (0%)	9 (4%)	7 (2%)	10 (2%)	19 (5%)	9 (1%)	57
Bovismorbificans	–	–	5 (5%)	1 (1%)	10 (4%)	–	19 (5%)	8 (2%)	–	12 (2%)	55
London	–	4 (20%)	–	–	3 (1%)	1 (0%)	8 (2%)	3 (1%)	14 (3%)	17 (3%)	50
Kedougou	–	1 (5%)	6 (6%)	2 (2%)	1 (0%)	6 (2%)	7 (2%)	13 (3%)	5 (1%)	9 (1%)	50
Orion	–	3 (15%)	5 (5%)	–	7 (3%)	6 (2%)	–	8 (2%)	12 (3%)	5 (1%)	46
Panama	–	–	1 (1%)	2 (2%)	9 (4%)	5 (2%)	3 (1%)	5 (1%)	7 (2%)	10 (2%)	42
Hadar	–	–	1 (1%)	1 (1%)	–	–	7 (2%)	18 (4%)	3 (1%)	1 (0%)	31
Enteritidis	–	–	1 (1%)	–	–	–	3 (1%)	1 (0%)	1 (0%)	12 (2%)	18
Other	–	4 (20%)	23 (25%)	13 (15%)	82 (33%)	48 (19%)	103 (28%)	70 (17%)	74 (18%)	117 (19%)	534
Totals	4	20	93	85	251	251	363	415	410	606	2,498

Percentage values are percent of total isolates for the year, rounded to nearest whole number. ^†^*Salmonella enterica* subsp. diarizonae.

**Figure 3:**
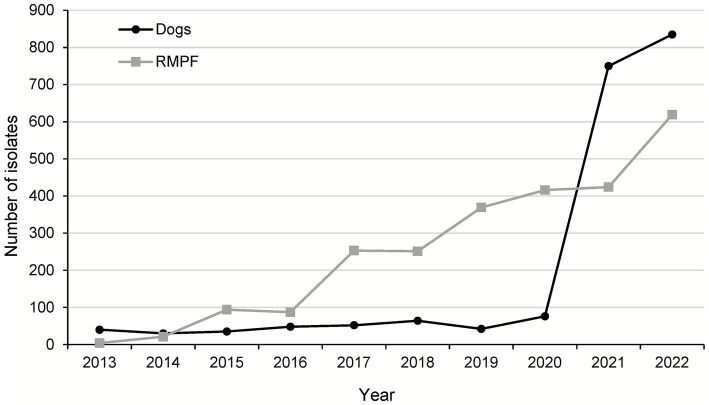
The number of *Salmonella* isolations from dogs and RMPF 2013–2022. Statutory reporting of *Salmonella* in dogs was introduced in February (England) and April (Scotland and Wales) 2021.

In the same decade there were 1,972 *Salmonella* isolates recorded from dogs. Figure 3 shows the almost 10-fold increase between 2020 (76 isolations) and 2021 (750 isolations) following changes to reporting rules. The most common serovars were *S.* Typhimurium, followed by *S.* Infantis and *S.* Dublin (Table 2). Over the decade, there was greater diversity in the dog data than RMPF with 93 different serovars (not including partial structures and rough strains) identified, although 38% of isolations were serovars of significant public health importance (Table 2). Similarly to RMPF, the frequently observed *S.* Typhimurium phage types included DT104, DT2 and DT193, the latter being most common among monophasic *S.* Typhimurium (Supplementary Table 1).

**Table 2:** *Salmonella* serovars isolated from dogs in Great Britain 2013–2022, top 20 named, listed in order of frequency.

Serovar	2013	2014	2015	2016	2017	2018	2019	2020	2021	2022	Totals
Typhimurium^†^	5 (13%)	6 (20%)	5 (14%)	12 (25%)	2 (4%)	20 (31%)	5 (12%)	12 (16%)	101 (13%)	115 (14%)	283
Infantis^†^	2 (5%)	1 (3%)	1 (3%)	2 (4%)	4 (8%)	1 (2%)	1 (2%)	5 (7%)	52 (7%)	105 (13%)	174
Dublin	4 (10%)	3 (10%)	6 (17%)	2 (4%)	6 (12%)	6 (9%)	2 (5%)	7 (9%)	64 (9%)	53 (6%)	153
Newport	2 (5%)	–	2 (6%)	2 (4%)	4 (8%)	3 (5%)	1 (2%)	6 (8%)	44 (6%)	52 (6%)	116
4,5,12:i:- ^†^	8 (20%)	3 (10%)	2 (6%)	8 (17%)	4 (8%)	1 (2%)	4 (10%)	7 (9%)	38 (5%)	39 (5%)	114
4,12:i:- ^†^	3 (8%)	–	5 (14%)	3 (6%)	6 (12%)	5 (8%)	6 (14%)	2 (3%)	36 (5%)	24 (3%)	90
Derby	2 (5%)	2 (7%)	–	–	3 (6%)	2 (3%)	–	2 (3%)	28 (4%)	51 (6%)	90
Enteritidis^†^	3 (8%)	2 (7%)	2 (6%)	2 (4%)	1 (2%)	1 (2%)	–	2 (3%)	26 (3%)	15 (2%)	54
Anatum	1 (3%)	1 (3%)	1 (3%)	2 (4%)	1 (2%)	1 (2%)	–	2 (3%)	20 (3%)	20 (2%)	49
Agona	–	–	2 (6%)	2 (4%)	–	–	–	1 (1%)	16 (2%)	27 (3%)	48
Montevideo	–	–	–	–	2 (4%)	1 (2%)	2 (5%)	2 (3%)	14 (2%)	25 (3%)	46
Bovismorbificans	–	–	–	2 (4%)	1 (–2%)	1 (2%)	–	1 (1%)	25 (3%)	9 (1%)	39
Indiana	–	–	–	–	2 (4%)	1 (2%)	–	2 (3%)	13 (2%)	20 (2%)	38
Kottbus	1 (3%)	1 (3%)	–	–	–	–	1 (2%)	2 (3%)	13 (2%)	20 (2%)	38
Agama	1 (3%)	1 (3%)	2 (6%)	–	2 (4%)	4 (6%)	2 (5%)	2 (3%)	13 (2%)	11 (1%)	38
Livingstone	–	1 (3%)	–	2 (4%)	–	–	–	–	12 (2%)	13 (2%)	28
Brandenburg	–	1 (3%)	–	–	–	–	–	3 (4%)	13 (2%)	10 (1%)	27
Mbandaka	1 (3%)	–	–	–	–	3 (5%)	2 (5%)	1 (1%)	6 (1%)	11 (1%)	24
Oslo	–	1 (3%)	–	–	4 (8%)	–	1 (2%)	2 (3%)	6 (1%)	10 (1%)	24
London	1 (3%)	–	–	–	–	–	–	–	7 (1%)	13 (2%)	21
Other	6 (15%)	7 (23%)	7 (20%)	9 (19%)	10 (19%)	14 (22%)	15 (36%)	15 (20%)	203 (27%)	193 (23%)	479
Totals	40	30	35	48	52	64	42	76	750	835	1972

Percentage values are percent of total isolates for the year, rounded to nearest whole number. ^†^Key serovars of public health importance.

#### 3.3.2 Antimicrobial resistance

Of the RMPF isolates that underwent antimicrobial sensitivity testing, 52% across all years were fully sensitive to all antimicrobials in the test panel. The observed resistance patterns showed large variations depending on serovar and phage type, with full sensitivity of individual serovars ranging from 0 to 100% (Table 3). Among those exhibiting antimicrobial resistance, multi-drug resistance (MDR, defined as resistance to four or more agents) was seen in a high proportion of some serovars, including monophasic *S.* Typhimurium (75 and 68% of *S.* 4,12:i:- and *S.* 4,5,12:i:- isolates, respectively), *S.* Infantis (30%) and *S.* Typhimurium (37%), but much less frequently in others, or in the case of *S.* Hadar or *S.* Dublin, not at all (Table 3).

**Table 3:** Details of antimicrobial resistance for serovars from raw meat pet food (RMPF) and dogs 2013–2022, including multi-drug resistance.

Serovar	Total isolates tested		Number, and proportion of isolates of that serovar from same source (RMPF or dogs)											
			Fully susceptible				Resistant to 1–3 antimicrobials				Multi-drug resistant^†^			
	RMPF	Dogs	RMPF		Dogs		RMPF		Dogs		RMPF		Dogs	
4,12:i:-	131	89	1	1%	4	4%	32	24%	19	21%	98	75%	66	73%
4,5,12:i:-	92	114	3	3%	1	1%	26	28%	23	20%	63	68%	90	79%
Bredeney	5	13	2	40%	10	77%	0	0%	2	15%	3	60%	1	8%
Rissen	13	15	5	38%	7	47%	2	15%	1	7%	6	46%	7	47%
Typhimurium	181	283	84	46%	159	56%	30	17%	42	15%	67	37%	82	29%
Infantis	122	174	73	60%	131	75%	13	11%	13	7%	36	30%	30	17%
Brandenburg	19	25	13	68%	18	72%	1	5%	7	28%	5	26%	0	0%
Kentucky	5	14	0	0%	5	36%	4	80%	1	7%	1	20%	8	57%
Panama	41	18	32	78%	18	100%	3	7%	0	0%	6	15%	0	0%
O-rough strains	20	16	9	45%	10	63%	9	45%	4	25%	2	10%	2	13%
Agona	12	46	9	75%	37	80%	2	17%	5	11%	1	8%	4	8%
Bovismorbificans	55	39	48	87%	35	90%	3	5%	4	10%	4	7%	0	0%
Newport	83	116	69	83%	101	87%	8	10%	13	11%	6	7%	2	2%
London	50	21	31	62%	21	100%	16	32%	0	0%	3	6%	0	0%
Anatum	19	48	12	63%	44	92%	6	32%	4	8%	1	5%	0	0%
Mbandaka	134	24	117	87%	22	92%	12	9%	2	8%	5	4%	0	0%
Derby	154	87	81	53%	64	74%	70	45%	23	26%	3	2%	0	0%
Indiana	338	38	104	31%	21	82%	226	67%	7	18%	8	2%	0	0%
Give	64	6	60	94%	5	83%	3	5%	1	17%	1	2%	0	0%
Dublin	57	152	56	98%	136	89%	1	2%	14	9%	0	0%	2	1%
Hadar	31	15	21	68%	5	33%	10	32%	9	60%	0	0%	1	7%
Livingstone	22	28	22	100%	27	96%	0	0%	0	0%	0	0%	1	4%
Stanley	18	14	17	94%	11	79%	1	6%	2	14%	0	0%	1	7%

^†^Resistance to at least four antimicrobial agents in the 16-drug panel. Serovars are listed in order of multi-drug resistance proportion among isolates from RMPF. Only serovars where 10 or more isolates from either source were tested are included.

Of the canine isolates, 68% across all years were fully sensitive to all tested antimicrobials. Monophasic variants of *S.* Typhimurium showed the highest frequencies of resistance, with fewer than 5% of isolates being fully sensitive (Table 3) and over 70% showing MDR.

### 3.4 Comparison between raw meat pet food and dogs in 2021 and 2022

Comparisons were restricted to isolates from 2021 and 2022, as the reporting requirements for dogs before this resulted in fewer submissions and uncertain additional bias(es) in the data.

#### 3.4.1 Serovars

**Figure 4:**
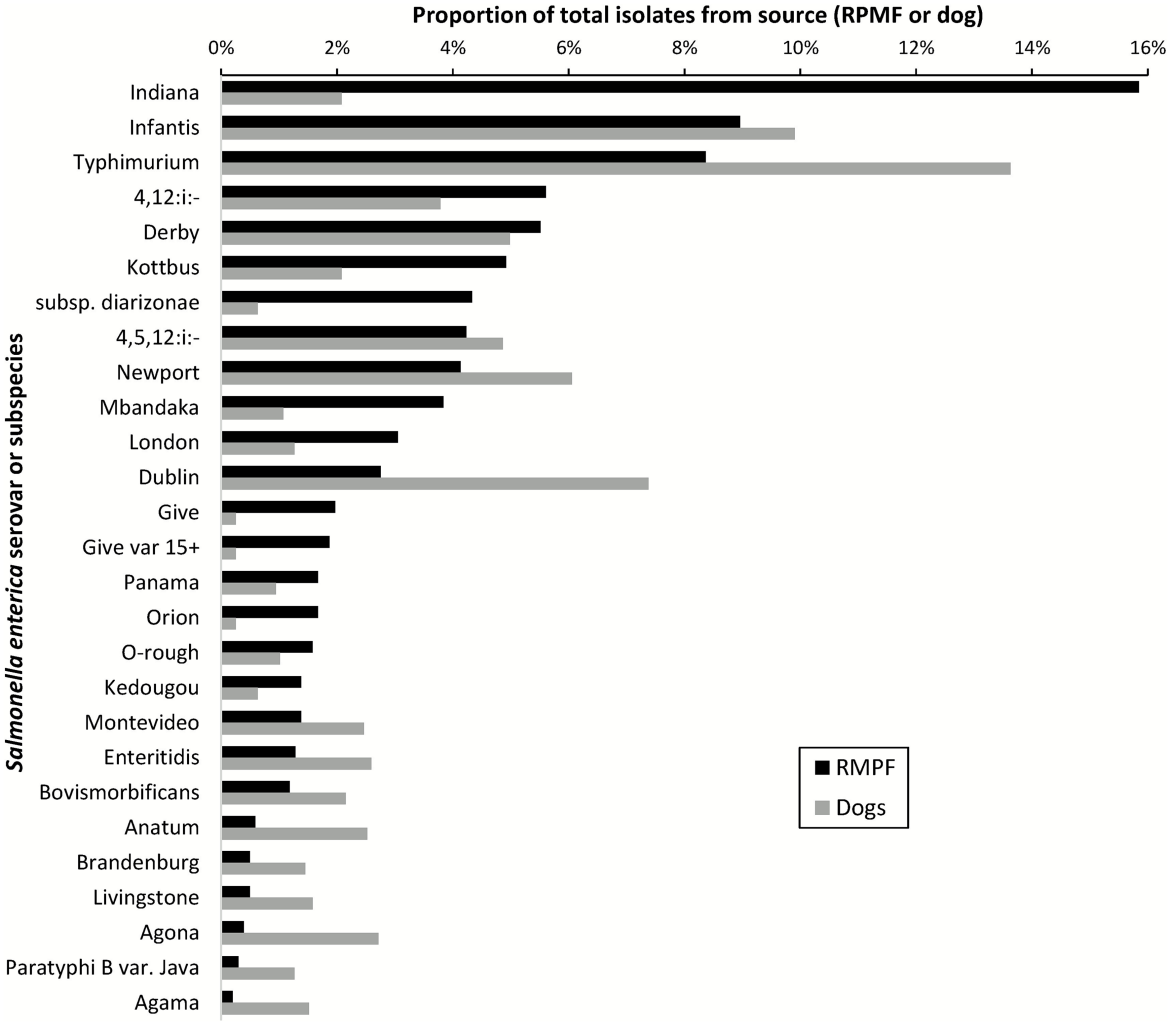
Comparison of serovar representation among 2,601 *Salmonella* isolates from raw meat pet food (RMPF, *n* = 1,016) and dogs (*n* = 1,585), years 2021–2022. Data comprises the top 20 serovars from each source, ordered by the frequency of isolation from RMPF.

The most common serovars from RMPF and from dogs overlapped considerably (compare Tables 1, 2; see Figure 4). In general, serovars commonly found in livestock such as *Salmonella* Typhimurium, monophasic *S.* Typhimurium, *S.* Infantis, *S.* Derby and *S.* Newport were frequently isolated from both RMPF and dogs. Some serovars were present in a comparatively higher proportion in the dog data compared with RMPF, for example *S.* Dublin at 7.4% in dogs vs. 2.8% in RMPF, and *S.* Typhimurium at 14% in dogs vs. 8% in RMPF. By contrast other serovars were more common in raw pet food than dogs, including *S.* Indiana (15.8% vs. 2.1%) and *S. enterica* subsp. *diarizonae* (4.3% vs. 0.6%). Among the serovars of significant public health importance, *S.* Typhimurium, monophasic *S.* Typhimurium and *S.* Infantis were all within the top 10 serovars isolated from both sources, whereas *S.* Enteritidis was ranked 9th in dogs but 20th in RMPF.

#### 3.4.2 Phage types of *S.* Typhimurium and monophasic variants

**Figure 5:**
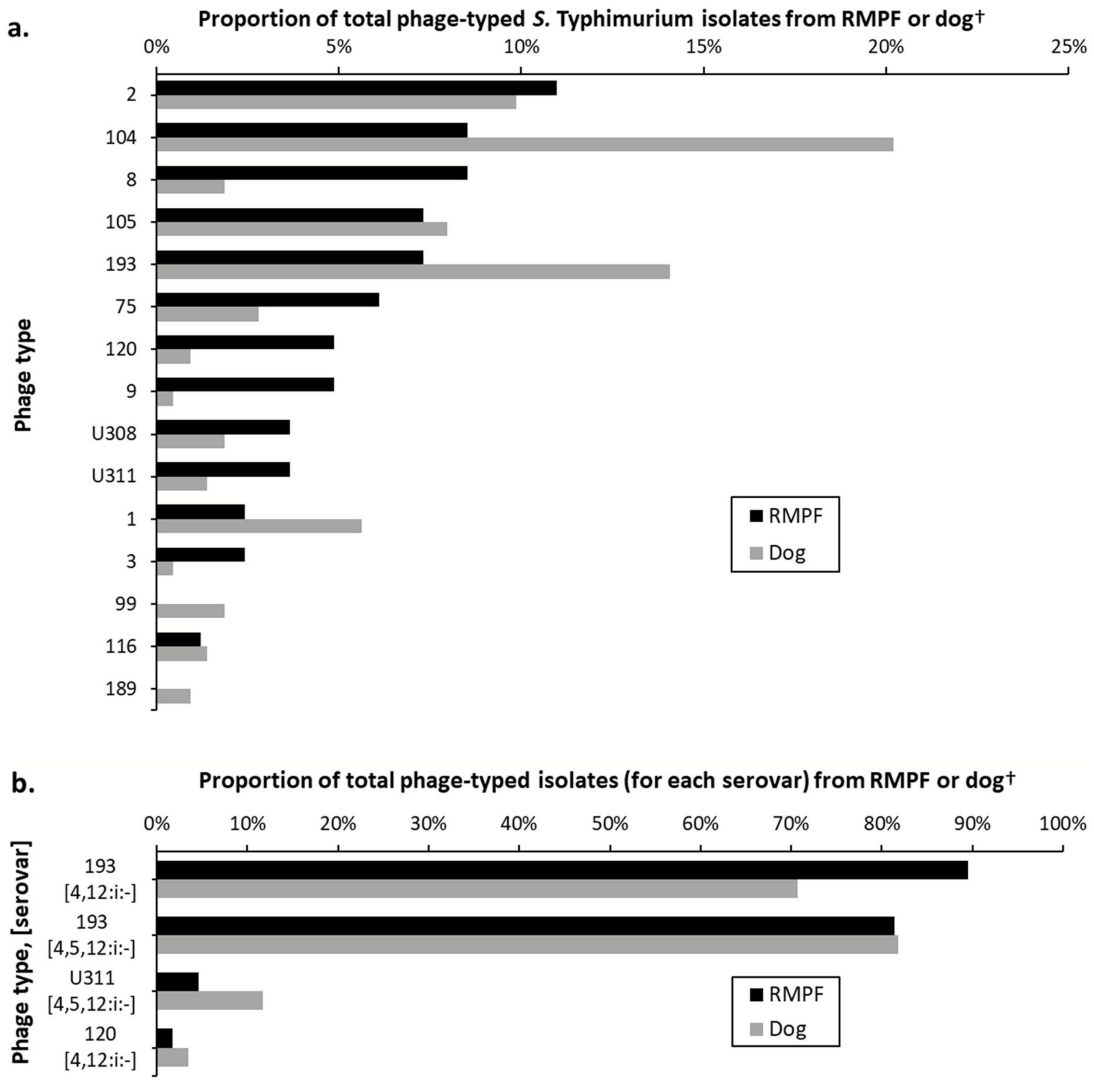
Comparison of phage-type representation among isolates of *Salmonella Typhimurium* **(a)** and its monophasic variants **(b)** from raw meat pet food (RMPF, *n* = 182) and dogs (*n* = 348), years 2021–2022. ^†^NOPT were excluded.

Frequencies of isolations overlapped substantially between RMPF and dogs (Figure 5; Supplementary Table 1). Considering the decade as a whole, DT2 and DT104 were the top-ranked *S.* Typhimurium phage types from both sources, although, DT193 became more frequent among the dog isolates accounting latterly for the second highest proportion of phage types after DT104. Among the monophasic *S.* Typhimurium strains, DT193 was dominant from both sources, accounting for over 70% of isolates in each case.

#### 3.4.3 Antimicrobial resistance

Over the decade as a whole, and for serovars commonly isolated from both RMPF and dogs (*S.* Typhimurium, monophasic *S.* Typhimurium, *S.* Infantis, *S.* Newport), there were similar proportions of MDR and fully sensitive isolates were recorded from both sources (Table 3). However, these proportions varied widely between serovars. The proportion of MDR isolates was highest among monophasic variants of *S.* Typhimurium, where between 68 and 79% of isolates from either source showed MDR. Multi-drug resistance was also frequently seen in *S.* Typhimurium and the less commonly isolated *S.* Rissen, from both RMPF and dogs.

##### 3.4.3.1 Resistance to individual antimicrobials

**Figure 6:**
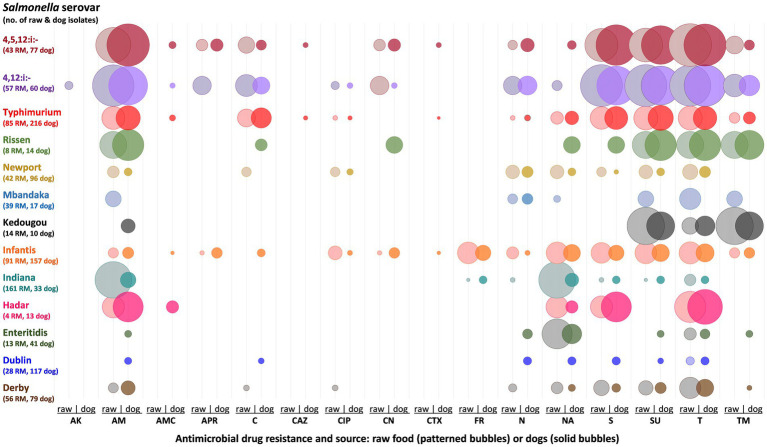
Bubble chart illustrating individual phenotypic resistances among *Salmonella* isolates from raw meat pet food and dogs, years 2021–22. Serovars included if of significant public health importance, or at least 10 isolates from either source showed resistance. Bubble area indicates the proportion of isolates expressing the resistance from that serovar and source combination, with non-zero proportions ranging from 0.5% (*S. Typhimurium*, dog, CTX resistance) to 93% (*S.* 4,5,12:i:-, food, T resistance). Degree of bubble overlap is a result of bubble size and in itself is not meaningful. AK, amikacin; AM, ampicillin; AMC, amoxicillin/clavulanic acid; APR, apramycin; C, chloramphenicol; CAZ, ceftazidime; CIP, ciprofloxacin; CN, gentamicin; CTX, cefotaxime; FR, furazolidone; N, neomycin; NA, nalidixic acid; S, streptomycin; SU, compound sulphonamides; T, tetracycline; TM, trimethoprim.

The proportions of isolates within a serovar that were resistant to individual antimicrobials were compared for RMPF and dogs for the period 2021 to 2022 (Figure 6). For monophasic variants of *S.* Typhimurium, resistance to certain drugs, such as ampicillin (AM), streptomycin (S), sulphonamide compounds (SU) and tetracycline (T), was present in a high (>70%) proportion of isolates from both sources. A similar pattern was seen in *S.* Typhimurium although with lower proportions (<30%) of isolates. For other serovars such as *S.* Infantis and *S.* Newport, resistances in RMPF to particular drugs were generally seen in low frequencies (<30%), and these were generally similar between RMPF and dogs. However, among *S.* Infantis isolates, resistances to certain antimicrobials (apramycin, ciprofloxacin, chloramphenicol, gentamicin and neomycin) were more variable between sources.

By contrast, some serovars presented distinctly different AMR frequencies between RMPF and dogs. *Salmonella* Mbandaka showed resistance to six different antimicrobials among RMPF isolates but only to neomycin from dogs. For *S.* Indiana, several resistances were seen at low frequency (less than 12% of isolates) from both sources, but ampicillin and nalidixic acid resistance were over five times more common from RMPF than from dogs. Serovars where resistances were seen from dogs but not RMPF, usually at low levels (<5%), include *S.* Dublin and *S.* Enteritidis. Certain serovars (*S.* Hadar, *S.* Rissen, *S.* Kedougou) were represented by few isolates and showed varied AMR phenotypes.

Considering medically important antimicrobial drug classes, resistance to either of the extended spectrum cephalosporins cefotaxime (CTX) or ceftazidime (CAZ), or to the fluoroquinolone ciprofloxacin (CIP) was rare. CIP resistance was seen from both sources and in several serovars in 2021–22 including *S.* Typhimurium, *S.* 4,12:i:-, *S.* Derby, *S.* Infantis, *S.* Newport and *S.* Kentucky. The highest frequency within a source/serovar combination in this period was 9.9% of *S.* Infantis isolates, from RMPF. All CIP resistance (across all 10 years) was seen in isolates that were resistant to at least one other antimicrobial, regardless of serovar.

In 2021–22 extended-spectrum cephalosporin resistance was seen only from dogs, and in only seven isolates each for CTX and CAZ. Combined resistance to both agents was seen in five cases: two isolates of *S.* Kentucky and single isolates of *S.* Typhimurium, *S*. 4,5,12:i:-, and *S*. Bredeney. In addition, a single isolate each of *S.* Infantis and *S.* Typhimurium showed resistance to CTX and CAZ, respectively. All these seven isolates were resistant to between one and six other antimicrobial drugs, including CIP resistance in one case (*S.* Kentucky). Resistance to amikacin was rare, with only two resistant isolates (of *S.* 4,12:i:-) from RMPF, both of which showed MDR.

##### 3.4.3.2 Patterns of multi-drug resistance

Comparing MDR profiles encountered since the start of statutory reporting of dog isolates in 2021, most isolates (77%) had resistance combinations that were seen both from dogs and RMPF (Table 4). The most common of these was tetravalent resistance (AM, S, SU, T), seen in 45% of dog and 55% of RMPF MDR isolates. Separately, the pentavalent resistance pattern (AM, S, SU, T plus chloramphenicol) was present in 25% of MDR dog isolates and 27% of MDR RMPF isolates, with or without additional resistances.

**Table 4:** MDR profiles shared between RMPF and dog sources, 2021–2022, with associated serovars and frequency.

Antimicrobial resistances		Serovar	RMPF	Dog
No. per isolate	Drug resistance profile^†^			
4	Am, C, Su, Sx, T	Typhimurium	1	4
	Am, Su, SxT, T	Rissen	3	4
	**Am, S, Su, T**	4,12:i:-	24	27
		4,5,12:i:-	18	42
		Typhimurium	1	5
5	**Am, C, S, Su, T** ^‡^	Typhimurium	3	18
	Am, N, S, Su, T	4,12:i:-	1	1
	Fr, NA, S, Su, T	Infantis	4	4
	Am, S, Su, SxT, T	4,12:i:-	1	1
		4,5,12:i:-	1	1
6	Am, Cip, N, NA, Su, T	Newport	2	2
	Am, C, NA, S, Su, T^‡^	Typhimurium	6	13
	Am, Fr, NA, S, Su, T	Infantis	2	1
	Cip, Fr, NA, S, Su, T	Infantis	8	2
7	Am, C, N, S, Su, SxT, T^‡^	4,12:i:-	3	7
	Apr, Cn, Fr, NA, S, Su, T	Infantis	1	9
	Fr, N, NA, S, Su, SxT, T	Infantis	3	1
9	Am, Apr, C, Cn, N, S, Su, SxT, T^‡^	4,5,12:i:-	1	2

^†^Am, ampicillin; Apr, apramycin; C, chloramphenicol; Cip, ciprofloxacin; Cn, gentamicin; Fr, furazolidone; N, neomycin; Na, nalidixic acid; S, streptomycin; Su, compound sulphonamides; SxT, sulphamethoxazole/trimethoprim; T, tetracycline. ^‡^Profile including pentavalent pattern. Core tetravalent and pentavalent resistance patterns are in bold.

## 4 Discussion

Most *Salmonella* isolates in the present data are from process control and monitoring samples in raw pet food plants. Heat-treated pet food is not free from *Salmonella* risk, especially if contamination exists in a post-heat-process stage of production (8, 31). However, surveillance in Great Britain consistently shows a higher frequency of *Salmonella* isolation from raw pet food compared with conventional compound pet food, despite much larger production quantities for the latter (32). This is consistent with the recent survey of frozen raw pet food at retail in the UK, which yielded viable *Salmonella* from 4.5% of raw meat diet products, but no *Salmonella* from cooked kibble (33), and also with recent surveys in Europe (34–41) and elsewhere (7, 42–46).

A wide range of serovars was identified in the present study. In dogs 93 serovars were detected, with *S.* Typhimurium, *S.* Infantis, *S*. Newport and *S*. Dublin the most frequently isolated. *S.* Typhimurium has previously been reported in dogs in Great Britain (47) and elsewhere (6, 48), reflecting its wide distribution and low host fidelity. *S*. Newport appears to be particulary prevalent among isolates from dogs in the USA (6, 49) while *S.* Infantis and *S*. Dublin have also been isolated from UK dogs (47). Diversity was lower in raw pet food, with 74 identified serovars, the most common being *S.* Indiana and *S.* Typhimurium. Both of these have also been isolated during surveys of raw pet food at retail in UK (33), suggesting that at least some of these sampled materials make it to retail. However, direct comparisons to existing studies are limited by differing timeframes, populations and data collection methods. Less common serovars may not be detected by snapshot prevalence surveys of raw pet food at retail because of a typically low frequency of *Salmonella* isolation (36, 46). There is also a notable paucity of comparable, published longitudinal surveillance data from other countries.

It is noteworthy that the proportions of the most common serovars reported from RMPF and dog surveillance in the present study were similar. It suggests, at least, a substantial overlap of original sources for these two sets of isolates, and potentially (also logically) transmission links between RMPF and dogs. By contrast, some serovars correlate rather poorly between the datasets, even allowing for different sampling biases for dogs compared to RMPF.

*Salmonella* Typhimurium was the most commonly isolated serovar of public health importance in the dataset and there was a broad correlation between the respective frequencies of definitive phage types isolated from RMPF and from dogs. The appearance of DT105 and DT75 among *S.* Typhimurium isolates from both sources from 2021 mirrors the emergence of both these phage types in Great Britain among ruminant livestock, and of DT75 in poultry (29). Among monophasic *S.* Typhimurium, the dominance of DT193 from both sources correlates with the pattern seen among isolates from British livestock generally (29).

By contrast, the frequency of *Salmonella* Indiana detection was substantially higher from RMPF than from dogs, consistent with other studies reporting a low frequency among dog isolates (6, 47, 49, 50). This serovar is strongly associated with duck farming in Great Britain (29, 51–53) and has previously been detected from raw pet food products containing duck (33). Indeed, duck is currently a popular ingredient in raw pet food in Great Britain, being present in over 70% of products reported by raw feeding owners in an online survey (33). The number of *S.* Indiana isolates from RMPF started to increase from 2017, a pattern not mirrored in livestock surveillance data from the period (29). However, in the absence of statutory *Salmonella* monitoring in ducks in Great Britan, sampling relies on discretionary testing of clinically healthy flocks, resulting in substantial year-to-year variation in sample numbers.

*S. enterica* subsp. *diarizonae* is another subtype showing a substantially higher frequency of isolation from RMPF than from dogs. This subspecies readily colonizes sheep, and is consistently the most frequently identified serovar from sheep in Great Britain (29, 54–56). It is known to have low pathogenic potential in humans, which may also be the case in dogs, as it is rarely isolated in either clinical or nonclinical samples from studies on this species (6, 47, 49, 57).

Sampling of the RMPF product is a process driven exercise, in part required for hygiene monitoring of feed production and in other cases forming part of due diligence and monitoring or investigation. By contrast, dog submissions are typically taken only when clinical salmonellosis is suspected, with testing influenced by factors such as access to veterinary care, owners’ resources, and illness severity. This means mild or subclinical infections caused by low-virulence serovars are far less likely to be sampled and therefore may be under-represented in dog isolates relative to dietary exposure. In contrast to *S.* Indiana and *S. enterica* subsp. *diarizonae*, the isolation frequencies of serovars Typhimurium and Dublin were substantially higher from dogs compared with RMPF. These last two are also among the small group of *Salmonella* serovars shown to host virulence plasmids that encode determinants of invasiveness (58). While we could not assess virulence gene distribution in the study, carriage of such plasmids may exacerbate clinical disease, and hence yield more submissions containing these serovars.

Comparison of AMR patterns provided additional evidence of potential associations between some serovars of *Salmonella* in RMPF and clinical isolates from dogs, in particular *S.* Typhimurium, monophasic *S.* Typhimurium, and *S.* Newport. Similarity of AMR patterns was less apparent for *S.* Infantis. Indeed, for many other serovars no similarities were evident. Certain serovars, particularly *S.* Dublin and *S.* Enteritidis, were not only present in higher proportions in dog isolates but also expressed antimicrobial resistances that were not seen from RMPF isolates. This suggests sources for many of these strains other than commercially prepared RMPF. While these might include fed raw items not from licensed and monitored raw pet food producers, there are several other routes through which dogs may encounter and ingest *Salmonella*.

In the subset of isolates from 2021 to 2022, there was considerable overlap between the MDR profiles from RMPF and dogs. This was particularly so among isolates of *S.* Typhimurium (both conventional biphasic strains and monophasic variants), *S.* Infantis and (in smaller numbers) serovars Newport and Rissen. This lends further weight to a hypothesized linkage between *Salmonella* in RMPF and dogs. The most common shared MDR patterns were tetravalent AM, S, SU, T and pentavalent AM, C, S, SU, T. These were also the most common MDR patterns observed among British livestock and animal feed stuffs during the same period, associated with *S.* Typhimurium DT104 and monophasic *S.* Typhimurium DT193, respectively (29).

Reassuringly, resistance to the medically important classes of fluoroquinolones and extended-spectrum cephalosporins were infrequent. Between 2021 and 2022 extended-spectrum cephalosporin resistance was recorded only from dogs. This might reflect a particular (unknown) route of exposure and/or a selection pressure from clinical use of beta-lactam antimicrobials in dogs. Fluoroquinolone resistance was more widespread, both among serovars and between dog and RMPF sources, but it was still present in only a small proportion of isolates. Nonetheless, fluoroquinalone and extended spectrum cephalosporin resistances were typically found in combination with resistances to other antimicrobial classes, and one MDR *S*. Kentucky isolate from 2022 was resistant to CTX, CAZ, and CIP, as well as to tetracycline. This last case may be an instance of a globally-disseminated MDR *S*. Kentucky strain (64). Such findings provide a warning against complacency in respect of AMR. Amikacin resistance was uncommon in the current study, and surveillance data in the USA shows similarly low levels in dogs (59). Amikacin is an important antimicrobial in human medicine to which resistance is also very uncommon among isolates from British livestock.

Feeding dogs raw meat diets is a risk factor for their shedding *Salmonella* in feces (7, 50, 60). However, despite the similarities (using several criteria) described here between *Salmonella* isolated from raw pet food and dogs, the presented data do not allow confident source attribution. There are several unascertained factors in the source data. These include the sample type for each RMPF isolate, which may be either finished product or ingredients. Many producers will reject contaminated batches based on *Salmonella* testing, so that much *Salmonella*-contaminated material does not make it to the raw food retail market. Also, the proportion of dogs yielding *Salmonella* that were fed RMPF is unknown. Furthermore, the most frequently observed *Salmonella* strains among RMPF and dogs are also common in the food chain more widely. This provides alternative potential routes for exposure of dogs to these *Salmonella* subgroups.

Nonetheless, there are valid concerns about possible transmission of *Salmonella* from RMPF to dogs, for which the present study provides some support, and also to the human population. Potential routes for a public health hazard include direct handling of RMPF and spread within the home on fomites or by pets (61, 62). Raw feeding has been associated with cases of clinical salmonellosis in dogs (13, 61, 63), and human salmonellosis cases have been reported in association with *Salmonella*-contaminated raw pet food in the USA (11). Furthermore, genomic analyses of *Salmonella* isolates from dog treats in Great Britain (14) and from dogs in the USA (49) have shown close relationships with contemporaneous human isolates. Further strategic work, refining and quantifying the *Salmonella* risk posed by RMPF to pets and humans, will benefit from these high-resolution genomic subtyping techniques to delineate transmission routes. The present mandatory surveillance regimes in the UK are well-placed to assist such studies.

## Data Availability

The data analyzed here are the results of both voluntary and statutory surveillance, reported to APHA. Raw (unaggregated) data are sensitive in nature due to potential for identification of individuals or companies. Requests to access these datasets should be directed to lucy.snow@apha.gov.uk.
