# Supplementary material for: Salmonella surveillance in raw pet food and dogs in Great Britain, 2013–2022
Source: Front Vet Sci. 2026 Feb 25;13:1750907. doi: 10.3389/fvets.2026.1750907 (PMC12975570; doi:10.3389/fvets.2026.1750907)
Supplement: Supplementary file 1 [file Table_1.docx]

Supplementary table 1: Phage types of Salmonella Typhimurium (ST), and monophasic variants, isolated from raw meat pet food and dogs in the UK, 2013 to 2022

| Serovar | Phage type | Proportion (%) of isolates from RPMF in the given year | | | | | | | | | |  | Proportion (%) of isolates from dogs in the given year | | | | | | | | | |  | All years | |
| --- | --- | --- | --- | --- | --- | --- | --- | --- | --- | --- | --- | --- | --- | --- | --- | --- | --- | --- | --- | --- | --- | --- | --- | --- | --- |
|  |  | ‘13 | ‘14 | ‘15 | ‘16 | ‘17 | ‘18 | ‘19 | ‘20 | ‘21 | ‘22 |  | ‘13 | ‘14 | ‘15 | ‘16 | ‘17 | ‘18 | ‘19 | ‘20 | ‘21 | ‘22 |  | RMPF | Dogs |
| ST | 104 |  |  |  |  | 50 | 13 | 50 | 44 | 9 | 8 |  |  | 17 | 20 | 58 | 50 | 15 | 20 | 8 | 18 | 22 |  | 19% | 20% |
|  | 2 |  |  |  |  |  |  | 8 | 17 | 11 | 10 |  |  | 17 | 40 | 25 | 50 | 20 |  | 25 | 13 | 7 |  | 9% | 12% |
|  | U302 |  |  | 80 |  |  | 10 |  |  | 3 |  |  | 20 | 17 | 20 | 17 |  | 10 |  |  |  |  |  | 7% | 2% |
|  | 193 |  |  |  |  | 25 |  |  | 8 | 6 | 8 |  |  |  | 20 |  |  | 10 | 20 | 0 | 16 | 12 |  | 5% | 12% |
|  | 8 |  |  |  |  |  |  |  |  |  | 14 |  |  |  |  |  |  |  |  |  | 3 | 1 |  | 4% | 1% |
|  | 105 |  |  |  |  |  |  |  |  | 3 | 10 |  |  |  |  |  |  |  |  |  | 6 | 10 |  | 3% | 6% |
|  | 75 |  |  |  |  |  |  |  |  |  | 10 |  |  |  |  |  |  |  |  |  |  | 5 |  | 3% | 2% |
|  | U288 |  |  |  | 100 |  | 10 |  | 3 | 3 |  |  |  |  |  |  |  |  |  | 8 | 1 |  |  | 3% | 0% |
|  | 1 |  |  |  |  | 25 |  |  |  | 6 |  |  |  |  |  |  |  | 5 |  |  | 3 | 8 |  | 2% | 5% |
|  | U308 |  |  |  |  |  |  |  |  | 9 |  |  |  |  |  |  |  |  |  | 8 | 4 |  |  | 2% | 2% |
|  | UNTY^†^ |  |  |  |  |  | 10 | 17 |  | 3 |  |  |  |  |  |  |  |  |  | 8 | 2 |  |  | 3% | 1% |
|  | Other^‡^ | 100 | 100 | 20 |  |  | 58 | 25 | 28 | 49 | 40 |  | 80 | 49 |  |  |  | 40 | 60 | 43 | 34 | 35 |  | 40% | 37% |
| *No. of ST isolates* | | *1* | *1* | *10* | *1* | *4* | *31* | *12* | *36* | *35* | *50* |  | *5* | *6* | *5* | *12* | *2* | *20* | *5* | *12* | *101* | *115* |  | *181* | *283* |
|  |  |  |  |  |  |  |  |  |  |  |  |  |  |  |  |  |  |  |  |  |  |  |  |  |  |
| 4,12:i:- | 193 |  | 100 | 57 | 50 | 67 | 20 | 37 | 63 | 47 | 53 |  | 27 |  | 71 | 18 | 40 | 50 | 40 | 22 | 31 | 29 |  | 49% | 31% |
|  | 120 |  |  | 14 | 6 |  | 20 |  |  | 3 |  |  |  |  |  |  |  | 17 |  |  | 1 | 2 |  | 3% | 1% |
|  | UNTY^†^ |  |  |  |  | 25 |  | 11 |  |  | 4 |  |  |  |  | 9 |  |  |  |  | 12 | 3 |  | 4% | 6% |
|  | other |  |  |  |  | 8 | 5 | 3 |  | 3 | 1 |  |  |  |  |  | 20 | 17 | 20 |  | 4 | 5 |  | 2% | 3% |
| 4,5,12:i:- | 193 |  |  | 14 | 39 |  | 50 | 46 | 37 | 44 | 31 |  | 64 | 33 | 29 | 64 | 40 |  | 40 | 67 | 43 | 49 |  | 35% | 46% |
|  | U311 |  |  |  |  |  |  |  |  | 3 | 1 |  |  |  |  |  |  | 17 |  |  | 5 | 8 |  | 1% | 5% |
|  | UNTY^†^ | 100 |  |  |  |  |  | 3 |  |  | 4 |  |  | 33 |  |  |  |  |  |  |  |  |  | 2% | 0% |
|  | Other^‡^ |  |  | 14 | 6 |  | 5 |  |  |  | 4 |  | 9 | 33 |  | 9 |  |  |  | 11 | 3 | 5 |  | 3% | 2% |
| *No. of monophasic ST isolates* | | *1* | *3* | *7* | *18* | *12* | *20* | *35* | *27* | *32* | *68* |  | *11* | *3* | *7* | *11* | *10* | *6* | *10* | *9* | *74* | *63* |  | *223* | *204* |

Specific phage types are listed in order of frequency of isolation from RMPF. ^†^UNTY = The culture did not react with any of the phages in the typing scheme. ^‡^ Isolates that were not tested or phage types with fewer than five isolates recorded from either RPMF or dogs are included under ‘Other’, unless there are more than five isolates recorded from the other source, in which case it gets its own entry.
